# Safety and Efficacy of Embolization with Microspheres in Chronic Refractory Inflammatory Shoulder Pain: A Pilot Monocentric Study on 15 Patients

**DOI:** 10.3390/biomedicines10040744

**Published:** 2022-03-22

**Authors:** Emeric Gremen, Julien Frandon, Gabriel Lateur, Mathieu Finas, Mathieu Rodière, Clément Horteur, Michaël Benassayag, Frédéric Thony, Régis Pailhe, Julien Ghelfi

**Affiliations:** 1 Faculty of Medecine, Grenoble-Alpes University, 38043 Grenoble, France; egremen@chu-grenoble.fr (E.G.); rpailhe@chu-grenoble.fr (R.P.); 2 Radiology Department, Grenoble Alpes University Hospital, 38043 Grenoble, France; mfinas@chu-grenoble.fr (M.F.); mrodiere@chu-grenoble.fr (M.R.); fthony@chu-grenoble.fr (F.T.); 3 Radiology Department, Nimes University Hospital, University of Montpellier, 30900 Nimes, France; julien.frandon@chu-nimes.fr; 4 Orthopaedic and Traumatology Surgery Department, Albertville Hospital, 73200 Albertville, France; g.lateur@gmail.com; 5 Department of Orthopaedic Surgery, Grenoble Alpes University Hospital, 38043 Grenoble, France; chorteur@chu-grenoble.fr; 6 Orthopaedic Surgery Department, Medipôle de Savoie Hospital, 73190 Challes-les-Eaux, France; m.benassayag@medipole-de-savoie.fr; 7 Institute of Advanced Biosciences, INSERM U1209, CNRS UMR 5309, 38043 Grenoble, France

**Keywords:** chronic shoulder pain, embolization, microspheres

## Abstract

Purpose: Musculoskeletal (MSK) embolization is emerging in tendinopathy treatment. The objective of this study was to assess the efficacy and safety of MSK embolization with microspheres in the treatment of chronic shoulder pain. Patients and methods: This retrospective monocentric study included patients with chronic shoulder pain resistant to 6 months or more of conventional therapies who were treated with MSK embolization between 2017 and 2021. Embolization was performed using calibrated 100–250 µm microspheres. Clinical success was defined as pain reduction, i.e., a decrease in the visual analogue scale (VAS) pain score of ≥50% at 3 months after MSK embolization as compared to baseline. Adverse events were collected. Results: Fifteen patients (11 women, 4 men) were included, with a median age of 50.3 years (IQR: 46.7–54.5). The median duration of symptoms was 26.6 months (20.6–39.8). The median VAS pain scores were 7.0 (7.0–8.0) at baseline, 6.0 (3.5–7.0) at 1 month, 5.0 (4.5–6.5) and 5.0 (3.0–7.4) at 3 months and 6 months (*p* = 0.002). Three patients (20%) reported clinical success at 3 months. Three patients experienced minor complications after embolization (paresthesia, *n* = 2; transient osteo-medullary edema, *n* = 1) and two patients had moderate complications (transient skin ischemia). Conclusion: MSK embolization with microspheres for treatment of refractory chronic shoulder pain showed moderate results in terms of clinical success and safety.

## 1. Introduction

Chronic shoulder pain is a global burden affecting between 4.7% and 46.7% of the adult population each year [1] and representing 26% of occupational injuries in France [2]. Painful shoulders include various pathological entities, often related to each other, among which are rotator cuff injuries (affecting approximately 30% of the population over 60 years old [3]) and retractile capsulitis (cumulated incidence between 8 and 10% of people of working age [4]). Physiopathology is complex and multifactorial for these two syndromes. A chronic inflammatory response with fibroblastic proliferation associated with angiogenesis has been described in several studies. It was shown to lead to joint fibrosis, often associated with synovitis, causing inflammation and painful neurogenic stimulation via non-myelinated sensory nerves. The treatment of these pathologies includes analgesic and non-steroidal anti-inflammatory therapies in association with an adapted rehabilitation, exclusion of favorable factors and, in some cases, coupled with corticoid injections [5]. About 30% of patients with tendinobursitis remain symptomatic after 6 months of conservative therapies [6]. Regarding retractile capsulitis, almost 30% of patients still reported symptoms after 7 years despite conservative therapies [7]. Some patients with severe and persistent symptoms are offered surgery with moderate results [8].

Recent studies have focused on destruction of the pathological loops of inflammation. Inflammation is at the origin of the development of neovascularization, feeding nerve growth and inducing chronic disabling pain [9,10]. These pathological non-myelinated sensory nerves are responsible for the maintenance of neovascularization via neurogenic stimuli [11], and neovascularization, in turn, maintains inflammation [12]. This relationship between tendon pain and vasculo-neural ingrowth was first explored by Alfredson et al. [13], who reported the efficacy of percutaneous sclerosis with polidocanol injection under US guidance in the specific area with neovessels outside the tendon. They showed good clinical results and signs of remodelling on sonography.

Based on this theory, musculoskeletal (MSK) embolization is emerging as one of the new analgesic therapeutic strategies for treating tendinopathies. It was recently reported in patients with tendinopathies, enthesopathies and retractile capsulitis of the shoulder with good safety and good clinical results [14,15,16,17]. An animal study on frozen shoulders showed that transcatheter arterial embolization decreased the number of neovessels and the number of mononuclear inflammatory cells as compared to the control group [18]. Clinical efficacy was also reported; running distance and speed were improved in the embolized group.

The major embolic agent reported in the literature for MSK embolization is an antibiotic solution, imipenem–cilastatin, administered in emulsion with contrast media, with temporary embolic properties after microcrystallization [12,19,20]. Although it showed promising results [14,15,16,17,21], the rapid emergence and dissemination of antibiotic-resistant microorganisms threaten adequate antibiotic coverage of infected patients [21] and limit its validation for daily use in embolization procedures. The causes of this problem are multifactorial, but the core issues are clear: the emergence of carbapenem resistance is highly correlated with selective pressure resulting from inappropriate use of these drugs [22]. Furthermore, the thrombogenic mechanism on neovessels caused by imipenem–cilastatin crystals is not yet understood.

Non-resorbable agents, such as microspheres, are used in daily practice in a wide range of embolization procedures, such as uterine fibroids [23], benign prostatic hyperplasia [24] and hemoptysis [25]. The use of microspheres in genicular artery embolization was recently reported in the management of symptomatic knee osteoarthritis refractory to medical therapies with good short-term results and no major complications [26,27]. The literature is still scarce on the use of microspheres in tendinopathy [28], with no dedicated study evaluating its effectiveness. The aim of this study was to report our first experience with non-resorbable microsphere embolization in patients with chronic shoulder pain and to evaluate its efficacy and safety.

## 2. Patients and Methods

### 2.1. Study Design and Patients

Our retrospective monocentric study was approved by a national institutional review board (CRM-2110-208). Before data collection, patients were informed about the study and could, in accordance with the local regulation for retrospective studies, oppose the use of their anonymized data for this research. Embolization was proposed for compassionate purposes to patients aged 18 years or older suffering from chronic inflammatory shoulder pain with restriction of movement and refractory to all conventional treatments (analgesics, non-steroidal anti-inflammatory drugs, corticosteroid injections, physiotherapy) for at least 6 months, after validation in a multidisciplinary meeting with an orthopedist, an algologist and a radiologist. All consecutive patients who underwent MSK embolization between June 2017 and September 2021 were included in the study. Patients were not included in the study if they had a complete rotator cuff tear, if they presented arthropathy secondary to chronic inflammatory rheumatism (rheumatoid arthritis, spondylarthritis) or microcrystalline rheumatism or had chronic renal insufficiency or hemostasis disorders. Pregnant women were not included. Patients who had MSK embolization for chronic shoulder pain with other embolization agents were also excluded.

### 2.2. Angiography and Embolization Techniques

For all patients included, the procedures were performed by one interventional radiologist (JG) with 6 years of experience. The procedures were scheduled on an outpatient basis in an angiographic room (Artis Zeego, Siemens, Erlangen, Germany). Under local anesthesia and ultrasound guidance, percutaneous arterial radial access was performed using a 4 F introducer sheath (Super Sheath; Medikit Co., Ltd, Tokyo, Japan). Digital subtraction angiography from the subclavian artery was performed with a 4 F angiographic catheter (multipurpose; Medikit Co., Ltd, Tokyo, Japan) to locate the arteries feeding the shoulder joint. The selective catheterization was performed with a 2.0 F microcatheter (Progreat, Terumo, Tokyo, Japan) to look at neovessels, i.e., abnormal vessels characterized on imaging as a tumor blush, as at arterial phase, with occasional early venous drainage.

**Figure 1:**
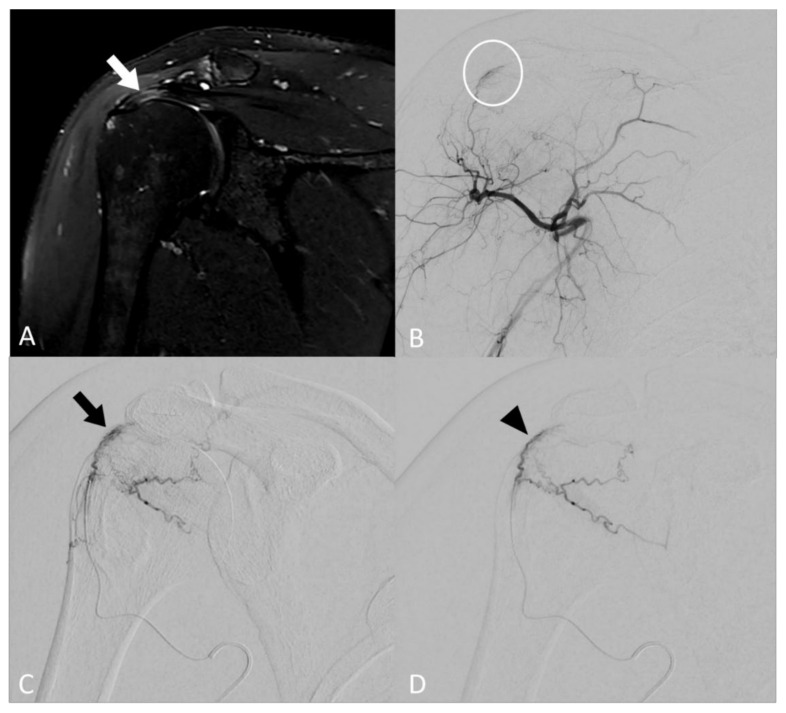
Image of a 46-year-old patient with rotator cuff tendinobursitis refractory to conventional treatment. (**A**) On baseline coronal T2 fat-sat MR imaging, injury appears as a hypersignal of supraspinatus tendon (white arrow). (**B**) The digital subtracted angiography (DSA) from the ostium of the posterior circumflex humeral artery confirmed pathological neovessel development in the area supplying the supraspinatus tendon (white circle). (**C**) DSA after selective microcatheterization of the pathological branch confirmed the “tumor-like” blush (black arrow). (**D**) DSA after embolization using microspheres demonstrated a decrease in the vascular blush (black arrowhead) that persisted, considering that the collaterals may be at risk of non-targeted embolization complications.

When neovessels were detected, embolization was carried out using calibrated microspheres of 100–250 µm (Embozene^®^, Boston Scientific Corporation, Malborough, MA, USA) diluted to the eightieth. The solution was loaded in a 1 ml syringe and injected 0.1 mL per 0.1 mL. A maximum volume of 4 mL of diluted particles (i.e., 0.05 mL of microspheres) solution was injected to limit off-targeted ischemic complications. Cutaneous collaterals were protected using an icepack on the skin. The patient related during the procedure whether the selective injection of iodine contrast reproduced the usual shoulder pain, thus reinforcing the operator to perform embolization in this area. The therapeutic strategy consisted in embolization of the neovessels, whilst carefully maintaining the larger vascular supply to off-target structures, such as the skin and bones. The disappearance of neovessels, i.e., the reduction of the tumor blush with a “dead tree” appearance, described in the literature [26] as “pruning embolization”, was considered the goal to be achieved with the embolization procedure. When collateral openings were reported during the embolization process, only a decrease of the blush was reached to avoid off-targeted embolization complications (Figure 1).

### 2.3. Study Objectives and Endpoints

The primary objective of the study was to evaluate the efficacy of MSK embolization in reducing pain. Clinical success was defined by a decrease of the pain ≥50%, measured on a visual analogue scale (VAS), as compared to pain measured at baseline [29]. The patients’ pain was evaluated both just before MSK embolization and at 3 months after the procedure.

The secondary objectives were to evaluate the technical success and safety of the MSK embolization procedure using non-resorbable microspheres to evaluate the minimum clinically important difference (MCID) of the procedure and to assess the evolution of MRI inflammatory signals before and after the procedure. Technical success was defined as embolization achieved for one or more arteries supplying neovessels, confirmed by angiography after the procedure. All complications related to MSK embolization that occurred between the MSK embolization procedure and the last patient follow-up consultation 6 months after the procedure were collected according to the Cardiovascular and Interventional Radiological Society of Europe (CIRSE) criteria [30]. The MCID was self-analyzed and comprised pain improvement at 3 months after the procedure (comparison of pain score at 3 months and at baseline and pain improvement (“Is pain less intense than before the procedure?” “Yes/No”)) and patient satisfaction with the procedure (“Yes/No”). Evaluation of the MRI inflammatory signals, i.e., MRI enhancement, was performed on T2 and T1 enhanced sequences before MSK embolization and 1 month after the procedure.

### 2.4. Statistical Analysis

Standard descriptive statistics were used for continuous quantitative variables presented as medians and interquartile ranges and for qualitative variables presented as numbers and percentages. A Wilcoxon signed-rank test was performed to compare pain VAS score before and after embolization. Fisher’s exact test was used to compare pain scores at baseline and 3 months for the MCID analysis. The level of statistical significance was set at 0.05 for all comparison tests. Analyses were performed on Excel^®^ (version 16.49) and using GraphPad Prism software (version 9.3).

## 3. Results

### 3.1. Patients’ Characteristics

There were 15 patients retrospectively included in the study. They were referred to our academic center and benefited from MSK embolization for chronic shoulder pain. Demographic data are summarized in Table 1. The study included 11 women (73%) and 4 men (28%); median age was 50.3 years old (IQR: 46.7–54.5). The patients’ main symptoms at baseline were nocturnal pain, reported by 13 patients (87%), and limitation of motion in 12 patients (80%). All patients reported that they felt limited in their daily activities. The median duration of symptoms was 26.6 months (IQR: 20.6–39.8). Six patients (40%) suffered from an occupational disease related to their shoulder injury. Nine patients (60%) had undergone surgery prior to embolization treatment. Six patients had adhesive capsulitis (40%), six had tendinobursitis (40%) and three had both (20%).

### 3.2. Angiographic Findings

The technical success was 100%, as MSK embolization by selective catheterization of at least one feeder artery with reduction of the vascular blush in angiography was achieved in all 15 patients (Table 2). The presence of neovessels was reported in all patients (100%). MSK embolization was performed by a homolateral radial approach in 14 patients (93%); for the last patient, MSK embolization was performed via a femoral arterial access because of a radial spasm. The median number of arteries treated per procedure was 2 (IQR: 2–3), with a total of 35 arteries treated. The targeted artery depended on the blush location and was the thoracoacromial artery in 8 patients (23%), the anterior circumflex humeral artery in 11 patients (31%), the posterior circumflex humeral artery in 9 patients (26%) and the scapular circumflex artery in 7 patients (20%). The median total volume of diluted microspheres solution was 3.0 mL (IQR: 2.4–3.5). The median procedure time was 106.0 min (IQR: 91.0–114.5), with a median scopy time of 32.1 minutes (IQR: 26.4–34.1).

### 3.3. Efficacy

**Figure 2:**
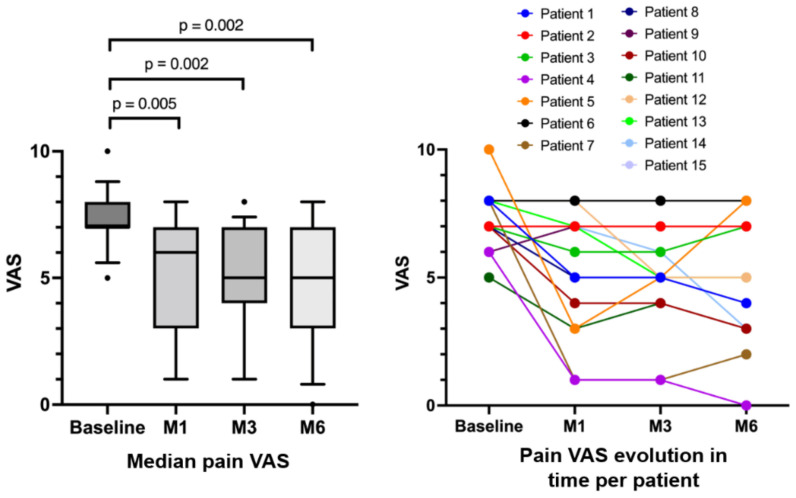
VAS at baseline and 1, 3 and 6 months after embolization; with median population comparison (**left**) and individual evolution per patient (**right**).

Clinical success, i.e., pain decrease ≥ 50% (VAS), was reported at 3 months in three patients (20%) (Figure 2). Among them, one patient reported recurrent symptoms at the 6-month follow-up visit. Among the other 12 patients, one patient reported a temporary pain increase at one month after embolization before he recovered the initial pain level at 3 months after the procedure. The median VAS pain scores were 7.0 (IQR: 7.0–8.0) at baseline, 6.0 (IQR: 3.5–7.0) at 1 month, 5.0 (IQR: 4.5–6.5) at 3 months and 5.0 (IQR: 3.0–7.4) at 6 months (*p* = 0.002). Among the patients who had initial nocturnal pain (*n* = 13/15), 10 reported an improvement (77%), whereas 3 patients did not report any difference (23%). Concerning range of motion, six patients (40%) reported an improved mobility 3 months after the procedure. No patient reported increased pain or decreased range of motion 3 months after the procedure. Concerning the MCID, three months after the procedure, four patients (27%) reported pain improvement and were satisfied with the procedure (*p* = 0.099).

### 3.4. Safety

**Figure 3:**
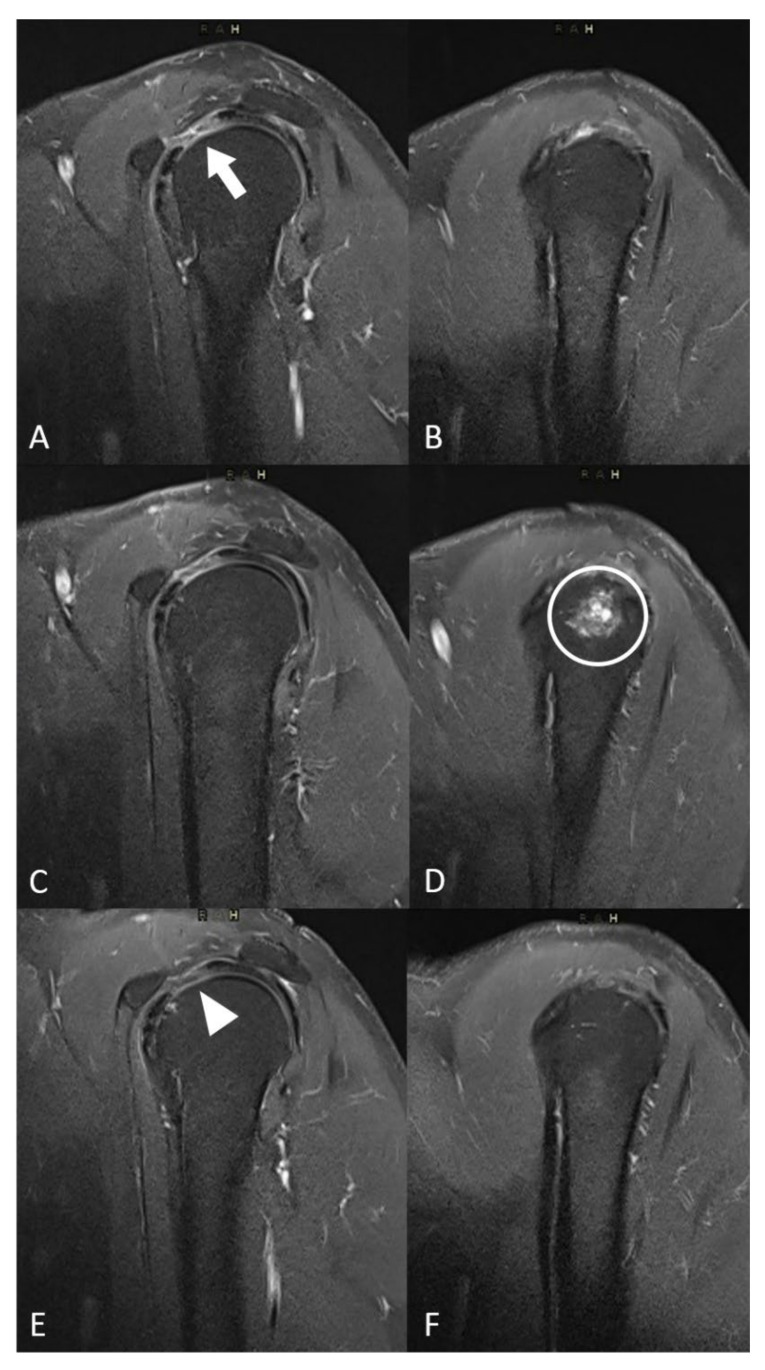
Evolution of MRI inflammatory signal and local complication. (**A**,**B**) Sagital T2 fat-sat MR images at baseline showed a hypersignal in the interval of rotator cuff (white arrow). (**C**,**D**) Sagital T2 fat-sat MR images one month after embolization showed a liquidian hypersignal in the humeral head (white circle) corresponding to focal oedema. (**E**,**F**) Sagital T2 fat-sat MR images 3 months after the procedure showed the decreased inflammatory signal in the interval of rotator cuff (white arrowhead) and the spontaneous disappearance of the humeral head edema.

Post-embolization syndrome, i.e., transient pain increase (around 10 days), was reported in eight patients (53%, Table 3). Two patients (13.3%) presented a moderate complication with transient skin ischemia managed with opioids and spontaneous recovery within 1 month. Three patients suffered from minor complications (transient paresthesia, *n* = 2 (13.3%)) and transient osteo-medullary edema (*n* = 1 (7.7%)) (Figure 3).

### 3.5. MRI Findings

Enhanced MR images before embolization were available for 13 patients (87%) and 1 patient had a non-enhanced MRI (including T2-weighted sequences) (Table 4). Angiographic blushes correlated with inflammatory signals on baseline MR images for 11 patients (85%). During the post-embolization period, 12 patients (80%) had enhanced MRI. A decrease in inflammatory abnormalities was reported on imaging in 9/12 patients (75%). The patient who presented osteo-medullary edema on MRI follow-up one month after embolization had spontaneous recovery, as shown on the 6-month control MRI with a decrease in the synovial inflammatory signal due to capsulitis (Figure 3).

## 4. Discussion

This study showed the moderate efficacy of MSK embolization with non-resorbable microspheres for treating chronic shoulder pain. Besides a technical success of 100%, moderate adverse events were reported with off-target embolization.

Our results showed a statistically significant reduction in pain, with a median VAS of 5.03 months after MSK embolization compared to 7.0 at baseline (*p* = 0.002). This effectiveness should be toned down by its clinical relevance: indeed, only 3 patients (20%) reported MSK embolization clinical success in terms of pain reduction, one of whom experienced symptom recurrence only 6 months after embolization. This is far less effective than MSK embolization for shoulder pain with imipenem–cilastatin, as reported by Okuno et al. [14] or Fernández Martínez et al. [16], with respective clinical success rates of 77% and 74%. Different factors may explain this difference and are discussed below.

The first is the embolization agent used. The mechanism of action of the imipenem–cilastatin solution used by other teams has not yet been elucidated. One hypothesis is that it may have pharmaco-mechanical emboligenic and anti-inflammatory properties [12] compared to the isolated mechanical action of the microspheres. Tendon repair is a complex phenomenon in which neoangiogenesis plays a significant role [31]. It also participates in pain with the development of neonerves [32]. To be effective, embolization should be temporary to destroy neonerves but should not affect neoangiogenesis in the long term. Embolization with particles, although ultra-selective, is a definitive embolization, unlike that of imipenem–cilastatin crystals, which allow temporary embolization via solubilization. Embolization using microspheres may be responsible for a prolonged ischemia, which may impact neoangiogenesis in the long term.

Second, the patients in our cohort were treated with MSK embolization with a compassionate purpose. They presented a heavy symptomatology that had evolved for a long period (median duration of symptoms was 26.6 months), much longer than those of cohorts in previously published studies (median of 7 [29] and 9 months [16]). In such a very long term, the processes of inflammation may certainly be anchored with irreversible lesions that may not be accessible with embolization. Moreover, in other cohorts with shorter symptom durations than in our population, it is possible that pain decrease and treatment efficacy may in fact be due to spontaneous positive evolution of the disease. The fact that a significant proportion of our patients (36%) reported an occupational disease may also interfere with the self-reported pain evaluation during follow-up.

Concerning the safety of the procedure, the occurrence of adverse events was significant, although we used the “pruning” technique described for geniculate artery embolization in osteoarthritis [26]. Transient ischemic cutaneous changes, MRI bone signal abnormalities and transient paresthesia were not reported after embolization with imipenem–cilastatin in shoulder embolization. On the contrary, transient paresthesia and change of skin color were described in genicular artery embolization using microspheres [26,33] with caliber microbeads of 100 µm or smaller. In 1999, a study reported that emulsion of the imipenem–cilastatin solution with iodine contrast resulted in small particles, around 10 to 70 μm [19]. As far as we know, the risk of off-target embolization increased with a smaller caliber of particles. It is thus more likely that the complications reported were due to the non-resorbable characteristic of the microspheres used than to the size of the beads. This opens discussion on the use of other temporary embolic agents in this field, such as gelfoam [34] or lipiodol. A study is currently evaluating the use of lipiodol in MSK embolization (NCT04733092)**.**

In our study, eight patients (53%) suffered a significant post-embolization syndrome (pain in the treated area, reversible in the next 10 days) that required analgesics. This was not reported in previous studies using imipenem–cilastatin. In genicular artery embolization with microbeads [33], it was also not reported. Diffuse synovitis was reported in a case of hemarthrosis and embolization with microspheres was not reported as painful despite the large synovial embolization volume [35]. The exact mechanism explaining this difference in pain between knee synovial/joint embolization and shoulder embolization has not yet been explained. It has been hypothesized that tendon injuries or bursitis may be more sensible to ischemia or that collaterals with extra synovial tissues are more frequent in shoulder vascular anatomy than in the knee.

The systematic use of enhanced MRI (T2-weighted coronal slices with fat signal saturation and T1-weighted slices without and after injection) may generate interesting data and possible correlations with angiographic serigraphs and thus increase the attention paid to tumor-like blushes during patient management. Some patients had angiographic blushes without contrast on pre-embolization MRI; however, T2-weighted hypersignals showing inflammation were often well correlated with the angiographic blushes.

Patients included in this study had symptoms lasting longer than 6 months and had failed standard treatments, including corticosteroid injections. Other approaches are currently being evaluated, although they are mostly oriented towards early damage for PRP injection [36] or do not seem to be more effective than corticoids for intra-articular hyaluronic acid injection [37]. Approaches using radiofrequency ablation of subscapular nerve have been reported, showing superiority over placebo treatment and physical therapy, but not over intra-articular injections of corticoids [38]. Studies on pulsed approaches on one or more nerves are also underway.

Our study has several limitations, one of which is its retrospective character. It was also monocentric, with a small sample, as it was a first experience in this indication, which limits the weight of our results. However, we believe that our safety results for MSK embolization with microspheres for chronic shoulder pain need to be reported, as it is currently performed in genicular arteries with very acceptable safety results, as described by early published reports [26,27,33,39]. In view of our safety results, we do not recommend the use of non-resorbable microspheres in MSK embolization for shoulder pain. However, as three patients (20%) reported significant improvement of their pain after MSK embolization, our results confirm the rising interest of such procedures for patients with chronic musculoskeletal injuries refractory to conservative therapies. Another limitation of our study is the absence of a control group and a comparison with other therapies, concordant with its pilot study design. Lastly, the primary objective of our study, pain reduction, was assessed as in many other studies, using a self-reported VAS scale. Assessment of pain evolution with more objective clinical evaluations would have been of interest.

## 5. Conclusions

MSK embolization with non-resorbable microspheres for chronic shoulder pain refractory to conventional therapies is feasible but associated with significant adverse events. Its efficiency seems to be lower than that of embolization with imipenem–cilastatin emulsion.

## Figures and Tables

**Table 1:** Patients’ characteristics at baseline.

Variables	*N* = 15
Age (years), median (IQR)	50.3 (46.7–54.5)
Gender, *n* (%)	
Female	11 (73)
Male	4 (27)
Nighttime pain, *n* (%)	13 (87)
Limitation of joint amplitude, *n* (%)	12 (80)
Limitation of daily life gestures, *n* (%)	15 (100)
Occupational disease, *n* (%)	6 (40)
Laterality, *n* (%)	
Right	10 (67)
Left	5 (33)
Surgical treatment before embolization, *n* (%)	9 (60)
Pathology type, *n* (%)	
Adhesive capsulitis	6 (40)
Tendinobursitis	6 (40)
Both	3 (20)
Duration of symptoms (months), median (IQR)	26.6 (20.6–39.8)

IQR: Interquartile range.

**Table 2:** Angiographic data.

Variables	*N* = 15
Technical success, *n* (%)	15 (100)
Number of treated arteries (by patient), median (IQR]	2 (2–3]
Targeted arteries, *n* (%)	
Thoracoacromial artery	8/35 (23)
Anterior circumflex humeral artery	11/35 (31)
Posterior circumflex humeral artery	9/35 (26)
Scapular circumflex artery	7/35 (20)
Volume of diluted microspheres injected (mL), median (IQR)	3.0 (2.4–3.5)
Homolateral radial access, *n* (%)	14 (93)
Procedure duration (min), median (IQR)	106.0 (91.0–114.5)
Scopy duration (min), median (IQR)	32.1 (26.4–34.1)
Dose (Gy.cm^2^), median (IQR)	15.6 (11.1–28.4)

IQR: Interquartile range.

**Table 3:** Complications reported after MSK embolization.

Variables	*N* = 15
Post-embolization syndrome, *n* (%)	8 (53%)
Grade I * complications, *n* (%)	
Transient paraesthesia	2 (13%)
Transient humeral osteo-medullary edema	1 (7%)
Grade II * complications, *n* (%)	
Transient skin necrosis	2 (13%)

* CIRSE classification system for complications.

**Table 4:** MRI findings.

Variables	*N* = 15
Baseline imaging modality, *n* (%)	
Injected MRI	13 (87%)
MRI without injection	1 (7%)
Arthroscanner	1 (7%)
Angiographic blushes correlated with baseline MRI contrast, *n* (%)	11 (85%)
MRI follow-up, *n* (%)	12/13 (92%)
MRI inflammatory signal decreased after embolization, *n* (%)	9/12 (75%)

## Data Availability

Data will be made available upon reasonable request to the corresponding author.
